# Parenting experiences of marriage immigrant women in South Korea during the COVID-19 pandemic: a descriptive phenomenological study

**DOI:** 10.4069/whn.2024.05.13

**Published:** 2024-06-28

**Authors:** Eunjung Ko, Hyun Kyoung Kim

**Affiliations:** 1 Department of Nursing, U1 University, Youngdong, Korea; 2 Department of Nursing, Kongju National University, Gongju, Korea

**Keywords:** COVID-19, Child rearing, Emigrants and immigrants, Qualitative research, Women

## Abstract

**Purpose:**

This study aimed to investigate the experiential meaning of child-rearing for marriage immigrant women in Korea in the context of the coronavirus disease 2019 (COVID-19) pandemic.

**Methods:**

Using the hermeneutic descriptive phenomenology framework developed by Colaizzi, 10 marriage immigrant women rearing preschool and school-age children were invited through purposive and snowball sampling from two multicultural support centers in Korea. The participants were rearing one or two children, and their original nationalities were Vietnamese, Japanese, Cambodian, and Chinese. Individual in-depth, face-to-face, semi-structured interviews were conducted from September 1 to November 30, 2021. We extracted significant statements from the transcripts, transformed these into abstract formulations, and organized them into theme clusters and themes to authentically capture the essence of the participants’ subjective experiences.

**Results:**

Four theme clusters with 14 themes were derived. The four theme clusters identified were “navigating child healthcare alone,” “guilt for not providing a social experience,” “worry about media-dependent parenting,” and “feelings of incompleteness and exclusion.” This study explored the perspectives of mothers raising children as marriage migrant women who experienced physical and emotional health crises due to the COVID-19 pandemic.

**Conclusion:**

The findings underscore that marriage immigrant women encountered heightened challenges in managing their children’s health and well-being during the COVID-19 pandemic due to linguistic and cultural barriers limiting access to healthcare and information. Additionally, these women experienced considerable emotional stress from perceived inadequacies in providing a holistic social and developmental environment for their children under extensive social restrictions.

## Introduction

Coronavirus disease 2019 (COVID-19), which emerged in 2019, has spread rapidly around the world. In March 2020, the World Health Organization declared it to be a global pandemic, and countries restricted movement [1]. Governments implemented social distancing, reducing economic activity, education, and care. The global economic downturn caused by the pandemic, unemployment, the decrease in educational opportunities, and deepening inequality between groups have left lasting scars on the economy and society as a whole [2]. More recently, breakthrough infections caused by mutated variants of the virus made daily life come to a halt again. People experienced feelings of helplessness, disconnection, fear, anxiety, stress, and boredom due to social isolation [3].

The number of migrants has dropped by 4.7% since the lockdown in South Korea. The decline in international migration has had an impact on changing perceptions of migrants. In South Korea, international marriage has become increasingly common since the 2000s under circumstances such as late marriage, low fertility, and an aging population among rural men. The number of marriage immigrants increased more than six-fold from 25,182 in 2001 to 169,633 in 2022 [4]. However, South Korean society is not socioculturally prepared to harmoniously integrate different races. The government’s multiculturalism policy is predominantly assimilationist, which is an obstacle to forming a sustainable multicultural society [5]. Marriage migrant women who immigrated to Korea for the purpose of marriage accounted for more than 80% of all immigrant women in 2018 [6]. The increase in the number of married immigrant women has led to an increase in the number of multicultural children, and this change is expected to affect the demographic structure of Korean society in the future [7]. The average age of multicultural children is 8.3 years old; 39.0% of these children are under 6 years old and 38.2% are aged 6 to 12 years—corresponding to elementary-school age—in 2018. The age distribution of multicultural children is concentrated in the preschool and school-age groups [6].

Marriage immigrant women with children experience many difficulties in raising and educating children due to insufficient Korean language knowledge and a low understanding of the Korean education system [8]. While the majority of Korean women are supported by their parental families, the mothers of married immigrant women are often unaware of the circumstances in Korea. The largest difficulty these mothers face in raising children is the lack of a network to support them when they are sick or busy [6]. A study on multicultural families abroad shows that the degree of acculturation of parents affects the family system and roles. Parents’ expectations for their children affect their positive development and social adaptation [9].

It is difficult for multicultural mothers who have difficulty understanding the Korean language to easily find or utilize the necessary information regarding COVID-19. The COVID-19 pandemic has exacerbated the difficulties faced by multicultural families that lack social support resources [10]. However, few studies have explored the parenting experiences of married migrant women in South Korea during the COVID-19 pandemic. The Korean Multicultural Families Survey showed that 24.4% of marriage migrant women had no intention of attending social gatherings in the near future, and there was no one with whom they could discuss their children’s education or who could take care of their children [4]. As the spread of COVID-19 and social distancing reduced interaction and communication with migrants, social prejudice and exclusion of migrants increased. Migrant women’s daily lives, labor, income, health, healthcare access, childcare, education, social relationships, and experiences of xenophobia have been severely affected during the pandemic [11]. In particular, the closure or reduced operation of elementary schools, daycare centers, kindergartens, care classes, and after-school programs due to COVID-19 has left a significant gap in care services. This has placed an additional burden of care on families, particularly affecting those with elementary and preschool-aged children. The difficulties faced by multicultural families that lack social support resources have been exacerbated [10]. Therefore, it is necessary to study the actual situation of marriage immigrant women who experience difficulties in raising preschool children in multicultural families.

Phenomenological research reveals the meaning of lived experiences by exploring the participants’ descriptions [12]. The research method of Colaizzi [13] enables researchers to grasp the meaning of humanly experienced phenomena through living experiences. Although a quantitative study on the parenting of multicultural families in Korea has been conducted [14], it predated the COVID-19 pandemic, and there is a lack of research on the parenting experiences of married immigrant women related to the pandemic situation. A recent study found that migrant women in Canada experienced fear, anxiety, stress, and a substantial burden of parenting [15]. Therefore, it can be seen that marriage immigrant women face serious psychological difficulties in the context of the COVID-19 pandemic. This study was conducted with the goal of deeply understanding the experiences of marriage immigrant women living in Korea and understanding the essential structure of those experiences [13].

The purpose of this study was to describe the meaning of experiences by understanding the perceptions and essence of child-rearing experienced in the life of marriage immigrant women in Korea in the context of the COVID-19 pandemic. The research question was “What is the meaning of the parenting experiences of marriage immigrant women with preschool and school-age children in the context of the COVID-19 pandemic in Korea?”

## Methods

**Ethics statement:** This study was approved by the Institutional Review Board of Kongju National University (KNU_IRB_2021-79). Informed consent was obtained from the participants.

### Design

This qualitative study applied the descriptive phenomenology research method of Colaizzi [13] to explore the essence of marriage immigrant women’s experience of raising children. This study was prepared according to the COREQ (Consolidated Criteria for Reporting Qualitative Studies) guidelines.

### Participants

Participants were marriage migrant women who could express their feelings and emotions in Korean. The inclusion criteria were (1) having a nationality other than Korean before marriage, and (2) having one or more preschool or school-age children after marriage with a Korean. The exclusion criteria were (1) women who moved to Korea after the outbreak of COVID-19, (2) women who lived abroad during the COVID-19 pandemic, (3) women who could not communicate in Korean, and (4) women who did not agree to participate in the study. The recruitment method began with a participant who was known through an acquaintance of the researchers. Then, snowball sampling was used, in which the first participant introduced to other participants. In total, 10 participants listened to the purpose and intention of the study and decided to participate in the study. Sampling was stopped when theoretical saturation was reached, meaning that the information provided by new participants became largely repetitive, and no new themes, patterns, or categories emerged from the data. Saturation was achieved when subsequent data collection efforts yielded no new thematic information, indicating that the dataset was comprehensive with respect to the research questions being investigated.

### Data collection procedure

Data were collected through in-depth face-to-face, semi-structured interviews with researchers from September 1, 2021 to November 30, 2021. Before setting the interview schedule, the purpose and method of the study were explained through individual phone calls, the interview schedule was planned, consent was obtained from the participants who agreed to participate in the study, and permission for recording was provided. In-depth face-to-face interviews were conducted at the desired time and place so that participants could feel comfortable. Each interview took about 1 or 2 hours, and interviews were conducted once or twice per participant. Participants were encouraged to think through the interview questions, and the researchers listened to them carefully. Two researchers transcribed the material recorded via mobile phone using handwriting and Naver’s automatic CLOVA program.

### Interview questions

The interviewer was a researcher, and the semi-structured questions for the interview were composed by the researchers in a pre-meeting and recorded in advance in the field notes. The grand tour question was “What experiences did you have with raising children during the pandemic?” This question was adjusted to use easy-to-understand language for participants. Probing questions included, “Please tell us about your experiences or feelings physically and emotionally”, “How did your children stop going to school or daycare due to COVID-19?”, “As children spent more time at home, how did your relationship with your child change?”, and “What experiences have you had with children receiving online education during COVID-19?” were used.

### Data analysis

Under Colaizzi’s framework for qualitative analysis [13] to return to the phenomenon itself, vividly described the subjective experiences that the participants experienced in the real world as they were revealed, the seven-step procedure of Morrow et al. [16] was used. (1) We read the participants’ statements repeatedly to familiarize ourselves with the data. (2) In order to identify significant statements, all sentences directly related to the phenomenon of parenting experience were found. (3) To create constructed meanings, we strove to avoid bias and carefully considered meaningful statements. (4) Among the meanings constructed for clustering themes, common contents were derived as themes. (5) We attempted to grasp the whole with a comprehensive description. (6) Themes were found using concise and meaning-dense language so that the essence of the phenomenon emerged. (7) Finally, two participants were asked to confirm whether the themes contained their experiences. The participant validation process was conducted to obtain a phenomenological perspective from natural attitudes. In addition, to obtain expert confirmation of the validity of the essential structure, opinions were elicited from three professors of women’s health nursing.

The three criteria of Cresswell [17] were applied to ensure research reliability. (1) The research procedures and questions were developed and conducted as a protocol. (2) Throughout the writing process, we referred to the transcripts of the interview and field notes. (3) We independently performed coding and discussed the consistency of coding in more than five meetings. Our team had an average of more than 10 years of experience in qualitative research, participation in domestic and international qualitative research meetings, review and editing of qualitative research, interview experience, and experience as educators. During the interview period spanning 5 months, we continued to interact and focused on discussing the direction of the research.

### Trustworthiness

Through an effort to return to the phenomenon itself through the process of bracketing the researchers’ understanding of the topic, we were able to look into the participants’ experiences without prejudice. Our prior understanding was that marriage migrant women would experience difficulties in raising children due to the lack of a child-rearing support system during the COVID-19 pandemic. These preconceived notions were recognized and a reflective thinking process was performed to avoid reflecting or inducing them in the interview. In order to respect the participants’ unique world of life, we respected and listened to the participants’ emotions and perceptions. We read the field notes repeatedly to understand the meaning of the participants’ experiences and shared and analyzed data by coding meaningful statements and constructing meanings, sub-themes, and themes using the Excel program. We also discussed the corresponding and differing issues in coding via face-to-face meetings. Discussion of inconsistencies continued until consistency was achieved with the provision of new meaning. If there was a difference between researchers, cyclical analysis was performed, in which the analyzed content was shown to the participants, who provided input that corrected the meaning.

## Results

### Characteristics of participants

The 10 participants ranged in age from 29 to 39 years, and their average age was 33.7 years. The average age of their children was 9.3 years (range, 4–15 years). All were married and had resided in Korea for an average of 10.5 years. Their nationalities before marriage were Vietnamese (five participants), Japanese (two participants), Cambodian (two participants), and Chinese (one participant). Nine participants obtained Korean citizenship after marriage and one was currently divorced. By occupation, five participants were office workers, three were housewives, and two were language support workers at a multicultural center. Only three participants had people to support them in child-rearing and half had completed high school (Table 1).

### Major theme clusters

Four theme clusters with 14 themes were derived. The theme clusters were “navigating child healthcare alone,” “guilt for not providing a social experience,” “worry about media-dependent parenting,” and “feelings of incompleteness and exclusion” (Table 2).

#### Navigating child healthcare alone

The participants noted that reduced outdoor activities led to heightened health risk factors, which were further exacerbated by challenges in dental care, declining vision, and difficulties in understanding medical prescriptions. This situation presented a complex array of health disparities and navigational burdens.

##### 1) Frustration with health risk

Participants expressed frustration over increased health risks as their children faced weight issues and dietary challenges due to restricted outdoor activities during the pandemic.

“Frankly, my son (12 years old) started gaining weight starting 6 months ago. That time he stayed at home. It’s been 6 months or more since online classes. He couldn’t go outside, so he was stressed out. Just... just at home. He ate a lot of instant food.” (Participant 2)

“My baby (7 years old) stays at home every day and doesn’t get sunlight. She needs to do a lot of outdoor activities. So, the baby did not eat well. Her weight gain was slow.” (Participant 4)

“He (11 years old) just got worse this year because of the coronavirus. He has to go around to exercise and get some air outside. He was only at home, so he gained weight and didn’t get enough vitamins. His glycated hemoglobin also became 7.8 and 8 points from 6.4 before. Ugh, I’m scared. Now (sigh)... It’s hard. Diabetes management also became difficult.” (Participant 5).

##### 2) Added burden of dental care

Added burdens in dental care due to COVID-19 restrictions at schools, which prohibited regular teeth brushing and use of gargles, led to deteriorating dental health and the emergence of issues such as tooth decay.

“He (11 years old) didn’t brush his teeth at school. He said, ‘don’t brush your teeth at school. because of the coronavirus.’ Because of the coronavirus, he didn’t even brush his teeth and didn’t wipe his mouth all day.” (Participant 5)

“She (12 years old) can’t even use a gargle at school. There’s something a little bit rotten with her teeth now. Because she can’t brush her teeth at school. The habit of brushing teeth was forbidden.” (Participant 6)

##### 3) Poor vision due to isolation

Participants reported that increased screen time due to isolation during the pandemic led to a deterioration in their children’s vision.

“I put the TV on or give him (7 years old) a cell phone a lot. I went to the eye clinic and heard from the doctor that my baby’s eyes got a little worse.” (Participant 4)

“Cell phone, cell phone. He (8 years old) has only been playing with the cell phone for over a year. Even if he studies, he is more interested in the cell phone. He doesn’t even want to go out to eat. Looking at the cell phone every day made his (11 years old) eyes hurt. When he goes to school now, he wears glasses. He can’t see the text on the blackboard. Kids have bad eyesight because of cell phones.” (Participant 5)

“I’m worried about this because of cell phone addiction. We didn’t talk to each other while looking at the smartphone and her (12 years old) eyesight got really bad within a year. She had to wear glasses.” (Participant 6).

##### 4) Coping with the medical situation alone

A significant challenge was managing their child(ren)’s medical conditions alone, especially under the constraints of the pandemic.

“My baby (8 years old) has diabetes and is not feeling well. If he gets the coronavirus, it will be really bad for him. I have a big job of injecting insulin, the doctor gives me everything. I am not Korean, and I don’t have much education and didn’t go to university. It is so hard because I am the only one who gives him his prescription. Really. These days, when the kids bother me just a little, I lose my temper and get angry like a lion.” (Participant 5)

#### Guilt for not providing a social experience

The limited opportunities to make friends and create childhood memories were noted to have significantly shaped the subjective sense of growing up in the pandemic, casting a shadow on the natural progression of social and emotional development.

##### 1) Pity for the child’s loneliness

Participants expressed a common feeling of sadness for children’s loneliness and concerns about the long-term impacts of these restrictions. Children’s inability to play freely, participate in regular school activities, and recognize friends during online classes heightened their sense of isolation and fear.

“Kids want to get together and play together. There is such a thing. I still couldn’t let him (12 years old) go. The kids were probably a little upset. But I explained the situation and how dangerous it was. He wants to play with his friends.” (Participant 2)

“My kid (7 years old) stayed at home for too long and was unfamiliar with his friends, so now he can’t get along with them again for too long. He is getting too scared. I think he has to meet a lot of friends again, talk and play. Isn’t it a little hard? I would like it if he could take off the mask and meet up with his friends of the same age and listen to classes and play together.” (Participant 4)

“He (13 years old) can’t see his friends even though school started. When he attends online classes in the morning, the teacher calls the names of the children attending. I ask my kid to learn his friends’ faces. Sociability, making friends is important. Now, even though he can meet friends, the kids don’t like to meet up anymore.” (Participant 6).

##### 2) Dissatisfaction about diminished opportunities to create childhood memories

The sentiment among participants reflected a profound dissatisfaction with the lost opportunities for children to make cherished memories due to the pandemic.

“He is now in grade 6, he can’t have either a graduation ceremony or a field trip due to the coronavirus. So many memories are gone, he didn’t even go out to play with the other kids. The time he spent in childhood is almost ended. Now, there’s not much left.” (Participant 6)

“Yes, it’s a pity that I haven’t arranged many activities with my children and I didn’t build up a lot of memories, but it’s too bad that [the opportunity] passed.” (Participant 7)

“The coronavirus has prohibited graduation ceremonies and taking pictures with friends. But these things don’t exist, so children are very disappointed. It’s not there, so it’s kind of sad.” (Participant 9)

#### Worry about media-dependent parenting

The increasing use of media during childcare led to parental ambivalence, balancing convenience against regret, and exacerbated the emotional dynamics between parents and children.

##### 1) Dehumanized digital-dependent parenting

Participants described resorting to digital-dependent parenting, where television and smartphones were frequently used as tools to occupy children during busy or difficult times.

“Housework takes a lot of time. But if the kids are around all the time, that doesn’t work either. So the kids keep watching TV again. I even showed him (5 years old) my mobile phone. If you give them a mobile phone, everything is quiet for several hours.” (Participant 3)

“I showed TV or mobile phone a lot to my babies. Since we can’t care for them all day, I did a lot of that. I’m worried—isn’t it bad for the eyes? I showed YouTube to my babies for a while when I was having a hard time.” (Participant 4)

##### 2) Ambivalence: convenience but regret

Participants expressed ambivalence about using television as a convenient but regrettable parenting tool, recognizing its utility for taking necessary breaks yet feeling uneasy about the potential negative effects on their children’s development.

“I showed TV to my child because I wanted to take a break. Even so, there’s a saying that TV isn’t good, it’s better not to watch it. When I was tired, I told her (6 years old) to watch TV. There are moments when I feel uncomfortable.” (Participant 8)

##### 3) Aggravation of emotions between parents and children

Participants described aggravated emotional dynamics in their relationship with their child(ren), largely due to conflicts over excessive use of digital devices such as mobile phones and computers.

“I argue with my child (12 years old) because they use mobile phones and computers a lot. Stop it! Phone! Sometimes I just take his phone. I was very angry with his father (who allowed that). No one in my family wears glasses. (Participant 2)

“Be sure to return the mobile phone by 8 pm. If I don’t remind him (13 years old) about it for a while, he doesn’t return it even if it’s late. If he watches the phone secretly, I get even more angry. He said he was talking to his friend in an angry voice, ‘Why can’t I just be a little late?’” (Participant 6)

“My kid (10 years old) always uses a laptop and plays phone games, and he does a lot of that. Because of that, he looks at the phone a lot. I’m very upset. But I can’t prevent it. I have to say it (stop) two or three times.” (Participant 10).

##### 4) Efforts to reduce overreliance on digital devices

Participants actively sought to mitigate their children’s reliance on digital devices by incorporating alternative activities

“I can’t keep watching the rest of the time, so I intentionally take him to run some errands. You have to kill time like this.” (Participant 2)

“My husband keeps buying used toys at the secondhand market every week. She (4 years old) can get a little bit of interest from them.” (Participant 3)

“My husband cares for the children and I think he shows them a lot of TV. So I like to do exercise at home, and I did something similar to yoga with my kids using a gym ball.” (Participant 7)

#### Feelings of incompleteness and exclusion

Participants’ narratives revealed a poignant struggle, where foods that lack the essence of nationality symbolize a loss of cultural identity, unfamiliarity with the Korean education system leads to a sense of disorientation as a primary caregiver, challenges exist in forming meaningful friendships as a mother, and it is necessary to rely on siblings, spouses, and neighbors in the shared journey of parenting.

##### 1) Not confident in assimilating culturally

Participants expressed a lack of confidence in culturally assimilating, particularly through cooking, as they struggled with preparing traditional Korean meals for their families.

“It’s so hard to cook. So I looked for Korean food on YouTube. So I found about 10 or 20 dishes that my husband and kids can eat. So I’m going to a cooking academy right now. My husband wanted me to pay more attention to the kids’ food.” (Participant 3).

“Cooking is the hardest thing. We have to change the menu every day. I think it’s the hardest. During COVID-19. You’re not good at Korean food either. I keep looking, searching, watching, and copying.” (Participant 4)

“It is not an easy task to provide food. Because it’s a dish with no name. I don’t know if it’s a Korean or Japanese dish. I ordered delivery from one of the side dish stores. I too have accumulated so much stress even with cooking that I can’t do it.” (Participant 7)

##### 2) Lack of experience in the education system

Participants highlighted their struggles with the education system, feeling underprepared and overwhelmed by the demands of assisting with their children’s homework and educational activities.

“The homework from kindergarten is to send me a study guide. I don’t even know what to write. I’m not good enough. I have to play the role of a teacher. I’m a foreign mom, which is why they give this to me too.” (Participant 4)

“School teachers upload their files and now they have to do a home correspondence or something like that. Even if they upload the files, I don’t know the contents. I can’t do it. The kids are doing their homework. I did it when I was in school in Vietnam and now it’s completely different. There’s no one to ask about anything. I still don’t know because I studied it the old way. The level is also very high, here in Korea.” (Participant 6)

##### 3) Socially disconnected as a mother

Participants expressed feelings of social disconnection as mothers, highlighting the challenges of managing childcare without personal time or opportunities for social interaction due to the pandemic restrictions.

“I have to spend time with my kids, so I don’t have personal time, I can’t meet other friends, and there’s no other way to relieve stress at that time. If it was the same as before, I would solve [my problems] with a friend. I went out and ate delicious food, but I couldn’t do that anymore, because I was socially disconnected.” (Participant 7)

“I honestly don’t have a place to leave my baby (6 years old). I have yet to meet Korean mothers who are close enough to leave my baby with. I am a little worried about who I can entrust with the baby. What should I do when I have to leave the child urgently? It’s not like we had opportunities to get to know each other like we did in the past, but it’s because we’ve become more careful with each other.” (Participant 8)

“I’m bored at home and I’m at home all the time. I can’t even meet friends, it’s so hard. It’s hard to work at home every day. When I was at home, I was frustrated because I was stressed and I scolded my kid a lot.” (Participant 9).

##### 4) Hard to parent without outside help due to the pandemic

Participants described significant challenges in parenting without outside help during the pandemic, particularly due to limited direct communication with schools and the need to manage children’s online classes while maintaining work commitments.

“I ask my husband or call the schoolteacher directly. I had a lot of difficulties asking for things.” (Participant 4)

“That’s an online class, I go to work every day. At home, only the kids (12 and 14 years old) take care of the online class. It was a little difficult for me for a few months, and I was worried too much. I was afraid I wouldn’t be able to keep up with their studies. My acquaintance at church comes here sometimes once a week to study together with the kids.” (Participant 9).

“I am a foreigner, notifications and text messages from school don’t reach me. I’m curious about a call from school. He (10 years old) tells me not to worry because his sister (11 years old) will take care of him.” (Participant 10)

## Discussion

This descriptive qualitative study explored the nature and structure of marriage immigrant women’s experiences while raising children during the COVID-19 pandemic using the phenomenological method of Colaizzi [13]. The experiences of raising school-age children in the early years of the COVID-19 pandemic were divided into four theme clusters and 14 themes.

The first theme cluster, “navigating child healthcare alone,” encompassed health risk factors due to increased consumption of instant food, obesity, the risk of diabetes, and a lack of activity, exercise, and sunlight exposure. The school closure restraint order, which was implemented as a strong administrative measure by the government to prevent the spread of COVID-19, resulted in reduced activity, increased screen time, weight gain due to irregular sleep and dietary intake, decreased cardiorespiratory function, and negative effects on mental health [18]. The lack of outside activities also affected the emotional health of children of immigrant families in Canada, increasing their fear and anxiety [15]. A decrease in visual acuity due to increased media use, changes in children’s lifestyle and habits, resulting in delayed sleep-wake times, and decreased outdoor activities are consistent with a prior study [15]. Children’s dental caries became more serious because of restrictions on the use of the bathroom at school, prohibiting brushing teeth at school, and using gargles rather than toothbrushes. Increasingly many participants have had to visit clinics or hospitals for the health management of their children. It was difficult for mothers to understand medical prescriptions for their children’s diseases. Marriage immigrant women have difficulties obtaining information from medical institutions due to language barriers, which makes it more challenging for them to encourage their children to engage in health-promoting health activities [18]. Both during the pandemic and in non-pandemic times, migrant women face significant challenges related to language barriers and cultural adaptation, impacting their ability to access healthcare, educational resources, and community support for themselves and their children. Navigating healthcare became even more challenging due to the pandemic-related restrictions, as healthcare systems were overwhelmed and information was rapidly changing. To prevent the future emergence of health problems, education and outreach should be strengthened to take into account linguistic and sociocultural limitations and low access to healthcare [17].

The second theme cluster, “guilt for not providing a social experience,” expressed low sociability due to reduced school attendance and the disappearance of opportunities to make childhood memories. It was reported that the lack of interactions with peers or teachers prolonged children’s tendency to refrain from going out due to COVID-19. The experiences of frustration and boredom can have a greater and longer-lasting psychological impact on children than physical problems [18]. Feelings of guilt and stress related to parenting responsibilities are common among migrant women, driven by concerns about providing adequately for their children’s social and educational needs in a new and often challenging environment. Sprang and Silman [19] reported psychosocial stress and lifestyle changes due to long-term reductions in outside activities, and it has been stated that a decrease in outside activities could negatively affect children’s physical and mental health, leading to a vicious cycle. Lockdowns and social distancing measures led to a significant reduction in school attendance and the loss of opportunities for children to interact with their peers, exacerbating feelings of guilt among migrant mothers. During COVID-19, the children of Latinx immigrants in the United States reported negative childhood experiences and toxic stress. There were restrictions on the use of schools’ social and emotional resources, and they were isolated because it was difficult to use social services [20]. Regular interactions with healthy adults are helpful for the social development of isolated children; thus, transcultural external resources other than parents are needed [20].

The third theme cluster, “worry about media-dependent parenting,” related to the time spent on childcare, increasing media use due to increased indoor activities, ambivalence, and aggravation of parent-child emotional dynamics. A previous study reported that children who spent more time alone at home due to school closures or reduced outdoor activities spent more time using electronic devices [18]. Migrant women often experience social isolation due to cultural differences, language barriers, and in some cases geographical separation from their families and support networks. The mothers felt sad about missing this important period for the children in Korean society [18]. A study not conducted among immigrants reported that parental domestic violence against children was higher in families with high parental stress, anxiety, economic burden, and parenting burden during COVID-19 [21]. During the pandemic, social hypervigilance, sensitivity, anxiety, and hostility increased, and discrimination against Asian minorities in particular increased [22]. At a time when social sensitivity to immigrants has increased, immigrant parents need more social support for raising their children because social stress is aggravated as their children spend more time indoors [19]. Various intervention programs, such as customized family unit interventions or psychological counseling for crisis management and family support, should be developed in consideration of each family’s functions and dynamics in order to increase families’ ability to overcome difficulties.

The fourth theme cluster was “feelings of incompleteness and exclusion.” Migration to Korea through marriage requires adaptation to the developmental task of marriage and environmental changes in the residential environment and culture as immigrants move away from their original families [6]. In particular, understanding Korean culture, cooking, and literacy to the degree necessary for raising children can be a crisis for immigrant women who have to adapt and raise children at the same time. These issues merit more attention because marriage immigrant women experience high parenting stress due to the lack of support from their original families as they are far away from their parents [8]. Koh and Koh [23] stated that marriage immigrant women experienced language barriers when using Korean medical services and had anxiety due to a lack of information about their children’s health status. In addition, negative psychological risk factors such as depression and anxiety due to COVID-19 increased social media exposure when family support was low [24]. With an increasing amount of time spent at home, the benefits of experiencing one’s native culture and spending meaningful time with one’s family were also reported [15]. Social support for marriage immigrant women is necessary because increased social support during a pandemic and a greater sense of control can significantly reduce the likelihood of excessive stress and the risk of child abuse [25]. The pandemic heightened the sense of isolation and exclusion among migrant women, not only from their communities due to social distancing measures but also from support systems that might have been accessible pre-pandemic. Support should be provided to establish a social safety net for married migrant women and an intergenerational experience transfer network for marriage migrants [26].

As discrimination against foreigners intensified during the pandemic, migrant women experienced a sense of disconnection due to social prejudice. Since negative views towards foreigners have increased through online media, timely and well-communicated education and public activities to promote sensitivity to human rights should be strengthened to avoid racism and discrimination [27]. Inclusiveness policies should be publicized through the media to prevent anti-immigrant sentiment and hostile feelings against immigrants in the post-pandemic society. Social support for marriage immigrant women is necessary because the more parents are provided with social support during a pandemic and the more they feel in control, the less likely they are to experience excessive stress and commit child abuse [25]. To promote equity in public health moving forward, immigrant communities’ participation in post-COVID-19 relief funding, social support, and vaccination policy processes should be prioritized [26].

This study provided deep insights into the difficulties of parenting experienced by marriage immigrant women living in Korea. Navigating child healthcare alone, guilt for not providing a social experience, worry about media-dependent parenting, and feelings of incompleteness were derived as theme clusters. This study will inform nursing interventions and family care policies for marriage immigrant women by describing their experiences in the COVID-19 context. This study explored the perspectives of mothers who raised children as marriage migrant women and experienced physical and emotional health crises due to the COVID-19 pandemic, both for themselves and for their children. This study also makes an important contribution by helping to understand the difficulties of marriage migrant women who feel that they are lacking as caregivers.

The limitations of this study are the fact that the study was conducted with mothers in only certain regions. The family is a basic unit of society, and further research is needed to specifically identify and measure aspects of the crisis in order to facilitate positive adaptations in a crisis situation. In addition, it is suggested that various intervention programs—such as customized family unit interventions or online psychological counseling for crisis management and family support—should be developed and implemented with consideration of each family’s functions and dynamics in order to increase the family’s ability to overcome difficulties.

## Figures and Tables

**Table 1.** Participants’ characteristics

Patient No.	Age (year)	Marital status	Number of children	Age of the child(ren) (year)	Length of residence in Korea (year)	Occupation	Nationality, original/current
1	38	Married	1	6	10	Insurance planner	Chinese/Korean
2	35	Married	2	13, 12	13	Service work	Vietnamese/Korean
3	31	Married	2	5,4	5	Housewife	Vietnamese/Korean
4	29	Married	1	7	7	Housewife	Vietnamese/Korean
5	32	Married	2	11, 8	11	Interpretation work	Cambodian/Korean
6	39	Married	2	15, 12	15	Interpretation work	Vietnamese/Korean
7	35	Married	2	8, 6	11	Nurse’s aide	Japanese/Korean
8	32	Married	1	6	7	Housewife	Japanese/Korean
9	35	Divorced	2	14, 12	14	Service work	Vietnamese/Korean
10	31	Second marriage	2	10, 11	12	Service work	Cambodian/Cambodian

**Table 2.** Themes and theme clusters derived from in-depth interviews

Themes	Theme clusters
Frustration with health risk	Navigating child healthcare alone
Added burden of dental care	
Poor vision due to isolation	
Coping with the medical situation alone	
Pity for the child’s loneliness	Guilt for not providing a social experience
Dissatisfaction about diminished opportunities to create childhood memories	
Dehumanized digital parenting	Worry about media-dependent parenting
Ambivalence: convenience but regret	
Aggravation of emotions between parents and children	
Efforts to reduce overreliance on digital devices	
Not confident in assimilating culturally	Feelings of incompleteness and exclusion
Lack of experience in the education system	
Socially disconnected as a mother	
Hard to parent without outside help due to the pandemic	
